# The Transcriptome of *Lutzomyia longipalpis* (Diptera: Psychodidae) Male Reproductive Organs

**DOI:** 10.1371/journal.pone.0034495

**Published:** 2012-04-05

**Authors:** Renata V. D. M. Azevedo, Denise B. S. Dias, Jorge A. C. Bretãs, Camila J. Mazzoni, Nataly A. Souza, Rodolpho M. Albano, Glauber Wagner, Alberto M. R. Davila, Alexandre A. Peixoto

**Affiliations:** 1 Instituto Oswaldo Cruz, FIOCRUZ, Rio de Janeiro, Rio de Janeiro, Brazil; 2 Departamento de Biologia Celular, Universidade do Estado do Rio de Janeiro, Rio de Janeiro, Rio de Janeiro, Brazil; 3 Institut für Zoo-und Wildtierforschung, Berlin, Germany; 4 Berlin Center for Genomics in Biodiversity Research, Berlin, Germany; 5 Departamento de Bioquímica, Universidade do Estado do Rio de Janeiro, Rio de Janeiro, Rio de Janeiro, Brazil; 6 Área de Ciências Biológicas e da Saúde, Universidade do Oeste de Santa Catarina, Joaçaba, Santa Catarina, Brazil; 7 Pólo de Biologia Computacional e Sistemas, FIOCRUZ, Rio de Janeiro, Rio de Janeiro, Brazil; University of Hyderabad, India

## Abstract

**Background:**

It has been suggested that genes involved in the reproductive biology of insect disease vectors are potential targets for future alternative methods of control. Little is known about the molecular biology of reproduction in phlebotomine sand flies and there is no information available concerning genes that are expressed in male reproductive organs of *Lutzomyia longipalpis*, the main vector of American visceral leishmaniasis and a species complex.

**Methods/Principal Findings:**

We generated 2678 high quality ESTs (“Expressed Sequence Tags”) of *L. longipalpis* male reproductive organs that were grouped in 1391 non-redundant sequences (1136 singlets and 255 clusters). BLAST analysis revealed that only 57% of these sequences share similarity with a *L. longipalpis* female EST database. Although no more than 36% of the non-redundant sequences showed similarity to protein sequences deposited in databases, more than half of them presented the best-match hits with mosquito genes. Gene ontology analysis identified subsets of genes involved in biological processes such as protein biosynthesis and DNA replication, which are probably associated with spermatogenesis. A number of non-redundant sequences were also identified as putative male reproductive gland proteins (mRGPs), also known as male accessory gland protein genes (Acps).

**Conclusions:**

The transcriptome analysis of *L. longipalpis* male reproductive organs is one step further in the study of the molecular basis of the reproductive biology of this important species complex. It has allowed the identification of genes potentially involved in spermatogenesis as well as putative mRGPs sequences, which have been studied in many insect species because of their effects on female post-mating behavior and physiology and their potential role in sexual selection and speciation. These data open a number of new avenues for further research in the molecular and evolutionary reproductive biology of sand flies.

## Introduction

*Lutzomyia longipalpis* (Lutz & Neiva, 1912) (Diptera: Psychodidae: Phlebotominae) is the main vector of American visceral leishmaniasis [1]–[2]. This sand fly is considered to be a complex of species [3]–[5], although no consensus has been reached upon the number and distribution of the different siblings [6]–[8]. In Brazil, the sibling species differ in their male copulation songs, pheromones and molecular markers. Nevertheless, the speciation process among the Brazilian populations is probably very recent and there is a paucity of markers with fixed differences allowing for a rapid identification of the different sibling species of the complex [9]–[10].

We still know relatively little about the molecular genetics of *L. longipalpis* and other sand flies, despite their medical importance. However, the construction and sequencing of cDNA libraries have been successfully employed for gene identification and characterization of gene expression profiles in whole insects [11] and in specific tissues such as salivary glands and midgut [12]–[15]. Transcriptome analyses of male reproductive organs have not yet been performed for *L. longipalpis*, although they may contribute to a better understanding of the molecular basis of sand fly reproductive biology. In addition, the sequences might provide new molecular markers to identify the different species of the complex. In this respect, genes expressed in male reproductive organs, such as accessory glands and testes, are particularly promising since they evolve rapidly [16]–[19].

Male accessory gland proteins (Acps), also known as male reproductive gland proteins (mRGPs), are major components of the seminal fluid which are transferred together with the sperm to the female during copulation, affecting the female's physiology and behavior [20]. These proteins and peptides belong to a number of different functional categories [21]–[22] and are known to be very important in insect fertilization because they are related to a variety of functions in female reproductive tracts. They are required to increase egg production, ovulation rate and sperm storage, as well as to reduce sexual receptivity. Moreover, they change feeding behavior and affect female longevity [23]. Indeed, insemination causes many changes in female gene expression [24], and in fact a very large number of female responses to mating can be also seen even when they mate with spermless males, highlighting the important role of male reproductive gland proteins [25].

Although sand flies lack a proper accessory gland, its role is probably played by the seminal vesicle. The insect seminal vesicle is normally a place to store sperm before its transfer to the female. In sand flies, this complex structured organ is formed by 3 distinct morphological compartments called A, B and C [26]–[27]. Compartment A probably works as the real seminal vesicle for sperm storage, whereas the compartments B and C are believed to elaborate and secret specific products, such as proteins and peptides, like in other insect accessory glands [28].

Different molecular and genetic tools coupled with bioinformatics have been used in the identification and analysis of Acps [29]. Ravi Ram & Wolfner [30] integrated results from several studies involving *Drosophila melanogaster* and identified 112 predicted Acp encoding genes. In the malaria vector *Anopheles gambiae*, at least 46 putative Acp genes have been reported [22]. Out of these Acps, 25 were designated as male reproductive tract-specific and 40% are homologues to *Drosophila* Acps. Among them figures the sex peptide, which is the principal modulator of female post-mating behavior in the fruit fly [31]. Interestingly enough, in *A. gambiae* the products of the male accessory glands transferred to the female reproductive tract form a coagulated mass called mating plug [32].

Acps belong to different classes of proteins that can be found not only in the reproductive tract but also in other insect organs. This fact associated to the fast evolutionary rate displayed by many of these genes makes the identification of their orthologues in other insects very difficult [16]–[19], [22]. In the case of disease vectors, in addition to *A. gambiae*, already mentioned above, proteins with similar biochemical features have also been found in *Aedes aegypti*, vector of yellow fever and dengue viruses [33]. These *A. aegypti* Acp-like proteins were called male reproductive gland proteins (mRGPs) because the analyses were carried out using accessory glands and ejaculatory ducts. Recently, Sirot et al [34] identified a number of *A. aegypti* male seminal fluid proteins, which were shown to be transferred to females during copulation. Many of those were homologous to *D. melanogaster* proteins, suggesting conservation of their function across Diptera.

In this paper we report an analysis of the transcriptome of *L. longipalpis* male reproductive organs and the identification of a number of putative male reproductive gland proteins (mRGPs). Our data not only constitutes a catalogue of expressed genes but also provides a molecular overview of the male reproductive system that might contribute to our understanding of the molecular and evolutionary biology of sand flies.

## Results and Discussion

### *L. longipalpis* male reproductive organ transcriptome

A total of 3068 clones were sequenced from a *L. longipalpis* male reproductive organs cDNA library to obtain 2678 (87.3%) high quality reads. Their clusterization resulted in 1391 non-redundant sequences, 255 clusters and 1136 singlets (Table 1). The non-redundant average sequence length was 409 bp, which is fairly short compared to the 605 bp found in the normalized cDNA library from *L. longipalpis* whole adult specimens [11]. This difference might be explained by a large number of singlets and the presence of small transcripts that seem to be commonly associated with reproductive organs in insects, as seen in *D. melanogaster* testis library ESTs (average length of 449 bp) [35]. On the other hand, it is important to notice that all reads under 350 bp were excluded from the whole sand fly cDNA library, which certainly increases the average read length [11]. All sequences (clusters plus singlets) generated in this work have been deposited in the GenBank EST database (accession numbers dbEST JK629524–JK632113, JK634704–JK634791).

**Table 1:** Summary of L. longipalpis cDNA library sequencing results.

Sequencing	Number
Sequenced ESTs	3068
High quality ESTs	2678

Clusterization	Number
Non-redundant sequences (singlets+clusters)	1391
Number of clusters	255
Number of ESTs in clusters	1542
Number of singlets	1136
Average non-redundant sequence size	409 bp
Average sequences/clusters	6.05

bp- base pair.

Among the 1391 non-redundant sequences, the majority contains between one and 10 ESTs which suggests a potential large diversity in this library. All non-redundant sequences were compared against a number of databases using different Blast flavors (Table 2 and Tables S1, S2, S3, S4 and S5). Interestingly, the most abundant clusters (number of reads >11) found in this cDNA library had no matches to proteins in the public databases with e-values below the cutoff used (1.0e-5), the same used in some other *L. longipalpis* EST papers [11], [14] and in all our subsequent analyses. More information about these abundant transcripts was obtained by searches of conserved domains using RPS-Blast and different databases (Table 2). Many of them (about 267 sequences) show similarity to cytochrome C or NADH dehydrogenase 6 domains. The presence of similar transcripts has been reported in some non-normalized libraries [14], [15].

**Table 2:** Results of cluster comparisons to different databases.

Program	Databases	Number of clusters with hits^a^
Blastx	Uniref-90.fasta	495 (36%)
	RefSeq_protein	484 (35%)
	*C.quinquefasciatus*.VectorBase (version J1.2)	446 (32%)
	*A.aegypti.VectorBase* (versionL1.2)	456 (33%)
	*A.gambiae. VectorBase* (version P3.6)	446 (32%)
	*D. melanogaster*-all-translation (r5.35 FlyBase)	464 (33%)
	Gene Ontology^b^	438 (31%)
tBlastx	*D. simulans* accessory glands library^c^	10 (1%)
	*D. melanogaster* testis library^d^	175 (13%)
	Drosophila ESTs	411 (30%)
	Acps/mRGP-Anopheles-Drosophila-Aedes^e^	23 (2%)
Blastn	Drosophila ESTs	145 (10%)
	*L. longipalpis* female -ESTs Database^f^	794 (57%)
	*D. melanogaster* testis library^d^	61 (4%)
Rpsblast	Cog	230 (17%)
	Cdd	558 (40%)
	Pfam	468 (34%)
	Kog	434 (31%)
	Smart	156 (11%)
	Prk	338 (24%)
	Tiger	136 (10%)

a - e-value cutoff: 1.0e-5 for all the Databases.

b - www.geneontology.org.

c - GenBank accession number BG642132 to BG642390.

d - Berkley Drosophila Genome Project (BGDP – www.fruitfly.org).

e - database constructed by us using sequences from Acps and putative Acps from *D. melanogaster* and *A. gambiae* and mRGP from *A. aegypti*.

f - Sanger Institute's *L. longipalpis* female EST database.

**Figure 1:**
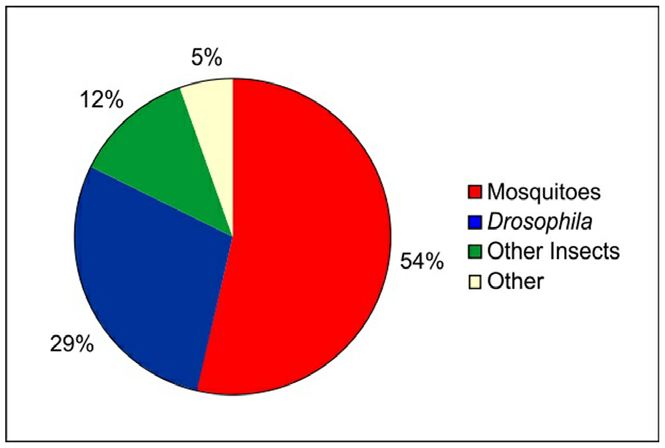
Distribution of the best Blastx matches for assembled *Lutzomyia longipalpis* ESTs. The ESTs were submitted to a search against the RefSeq_protein database (NCBI). The e-value cutoff was 1.0e-5.

Among the sequences that yielded hits to potential orthologues, 54% of the best matches were found against mosquitoes (*A. aegypti*, *A. gambiae* and *Culex quinquefasciatus*), followed by 29% against Drosophila (Figure 1, Table S2).

A list of the hits obtained with some of the other databases is available in Tables S3, S4 and S5, where the EST sequences are classified according to their best hits when compared with sequences from other insect species. Although small variations have been observed among the results using different protein databases, no more than 36% of the *L. longipalpis* male reproductive organs ESTs showed significant similarity to any of the databases (e.g. RefSeq_protein and Uniref-90.fasta). In Drosophila, 47% of the ESTs in a male accessory gland library presented no similarity to other fruit fly sequences in GenBank [16]. A comparison to the available *L. longipalpis* female EST database yielded hits with only 57% of our clusters (Table 2). The large proportion of ESTs with no hits with the *L. longipalpis* female database suggests that many are potential male specific genes.

### Gene Ontology classification

**Figure 2:**
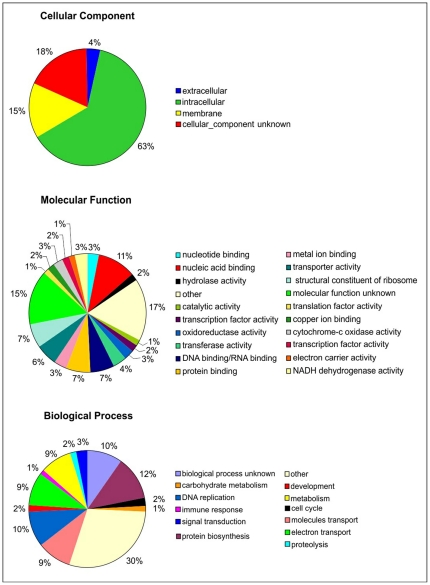
Classification of ESTs in Gene Ontology category. The ESTs of *L. longipalpis* were submitted to a search against the three categories of Gene Ontology (NCBI). The e-value cutoff was 1.0e-5.

We obtained Gene Ontology (GO) classifications for 438 (31%) non-redundant sequences in three ontology domains: cellular component, molecular function and biological process. An overall view of the distribution of the sequences in the three ontologies can be seen in figure 2. In the cellular component category there is a clear prevalence of intracellular sequences while the second most abundant category is of unknown function. Regarding the molecular function classification, the main groups are involved in nucleic acid and protein binding and transporter activity. A large fraction of the sequences has unknown molecular function. In addition, several sequences with less than 1% similarity were grouped into the class designated as “other”.

In the biological process category the prevailing groups are associated to DNA replication, protein biosynthesis, metabolism and transport of molecules and electrons. It is known that higher levels of protein synthesis have been observed in post-meiotic stages during spermatogenesis [35].

### ESTs encoding putative Male Reproductive Gland Proteins (mRGPs)

We identified 14 ESTs encoding putative mRGPs or Acps (Table 3). In an initial analysis, neither mRGP nor Acp homologues were identified for *L. longipalpis* using Blastx searches against usual protein databases (e.g. Refseq and Uniref-90), in accordance to what has been observed for *A. gambiae* [22]. To constrain the search, a local customized database with available insect primary mRGP/Acp peptide sequences was created (see Methods).

**Table 3:** Putative *L. longipalpis* mRGPs.

Cluster Name	N	Cluster length	Acps DB Homology	e-value	Predicted PTN class	Diptera DB Homology	e-value	Predicted PTN class
RAAPBAR005E03*	1	514	AGAP007049	2.0e-18	Defensin	AGAP007049	2.0e-14	Defensin
RAAPBAR022E08*	2	598	AAEL013279	2.0e-75	Cyclophilin	AAEL013279	9.0e-62	Cyclophilin
RAAPBAR013A01	1	299	AAEL001713	4.0e-15	Fibrinogen/Fibronectin	AAEL009723	5.0e-41	Fibrinogen/Fibronectin
RAAPBAR018E11	2	552	AAEL007063	5.0e-19	Lipase	AAEL007044	5.0e-23	Lipase
RAAPBAR031D02	2	646	AAEL007063	3.0e-37	Lipase	AGAP004449	9.0e-48	Lipase
RAAPBAR013H06*	1	687	COEBE4D	5.0e-23	COEBE4D	AGAP005370	9.0e-33	COEBE4D
RAAPBAR030E12	2	642	AAEL006576	2.0e-13	Protease	AGAP004148	8.0e-32	CLIP-domain Serine Protease
RAAPBAR008G04	1	487	AAEL013449	1.0e-11	Metalloprotease	AAEL001330	4.0e-14	Zinc Metalloprotease
RAAPBAR022F08	1	731	AAEL013449	1.0e-35	Protease	AGAP010764	5.0e-47	Zinc-dependent metalloprotease
RAAPBAR023H02*	1	619	AAEL000551	6.0e-22	Protease inhibitor	AAEL000551	3.0e-25	Protease inhibitor
RAAPBAR022H02	2	708	AAEL009239	8.0e-18	Crisp	AAEL010269	3.0e-21	Venom Allergen
RAAPBAR005C10*	1	586	AAEL007777	2.0e-22	ATPase synthase	AAEL007777	4.0e-17	ATPase synthase
RAAPBAR020D12	1	585	CG6988	2.0e-09	Thioredoxin	AAEL010777	2.0e-38	Thioredoxin
RAAPBAR011H02	2	513	AAEL000641	3.0e-10	Protein folding	AGAP007201	5.0e-41	Thioredoxin

**N-** Number of reads. **DB**- Database. PTN- Protein. COEBE4D- carboxylesterase, beta esterase. Crisp- Cysteine-rich secreted proteins.

* ESTs that have yielded best matches to mRGPs/Acps from protein databases (three against *A. aegypti* and two against *A. gambiae*). AGAP00 Sequences come from AgamP3.6_vectorbase and AAEL0 Sequences come from AaegL1.2_vetorbase.

Blast searches were performed in two steps, first the *L. longipalpis* cDNA library was used for a search against the customized mRGP/Acp database in order to identify potential mRGP/Acp orthologues (Table S6). In addition, the *L. longipalpis* sequences that yielded matches to mRGPs/Acps were subsequently tested against complete protein databases of the insect species that presented the best e-values in the first search (*D. melanogaster*, *A. aegypti* or *A. gambiae*). Five out of the fourteen initially identified ESTs (in asterisks) yielded best matches to mRGPs/Acps from the species-specific protein databases (three against *A. aegypti* and two against *A. gambiae*). The remaining nine ESTs presented best matches to proteins belonging to the same families as some of the known mRGPs/Acps (Table 3).

Three (RAAPBAR022E08, RAAPBAR022F08 and RAAPBAR018E11) out of the 14 sequences identified as potential *L. longipalpis* mRGPs showed a high probability of being secreted proteins as determined by the Signal P program. The presence of the signal peptide is an indirect criterion to identify many of the Acps and mRGPs [30]. For some of the remaining sequences, the alignment with homologues from other insects revealed that, the N terminal region is absent and, therefore, lacking the putative signal peptide. In addition, it must be noted that the absence of a putative signal peptide in a complete sequence does not exclude the possibility of a polypeptide being a mRGP/Acp, since some of these proteins found in other insects do not have a signal sequence [22], [33], [34].

As observed in other insects the putative *L. longipalpis* mRGPs belong to diverse classes of proteins (Table 3). Among these ESTs, four are probably involved with proteolysis (one serine protease, two metalloproteases and one protease inhibitor), two with immunity, two in the redox metabolism (thioredoxins), one is associated with coagulation, one is an ATP synthase, two are lipases, one is a carboxyl esterase (COEBE4D) and one is a cysteine-rich secretory protein (CRISP). A more detailed description of these sequences is presented below.

### *L. longipalpis* ESTs related to immunity

The EST RAAPBAR005E03 is essentially identical to an EST (AM091821) from a *L. longipalpis* female cDNA library. As the male sequence is incomplete a contig of both sequences (RAAPBAR005E03/AM091821) was used in analyses. This sequence shows similarity with an Acp of *A. gambiae*, which has a β-defensin domain (AGAP007049), and its homologues in *A. aegypti* (AAEL009861), *C. quinquefasciatus* (V10860) and *D. melanogaster* (CG10433). Defensins are antimicrobial peptides involved in insect immune response against bacteria, viruses and protozoa. As the female reproductive tract is rich in pathogens introduced during the mating process so antimicrobial peptides could be involved in the success of fertilization because they may protect the seminal fluid or the female reproductive tract from microbial infections [36]–[37].

**Figure 3:**
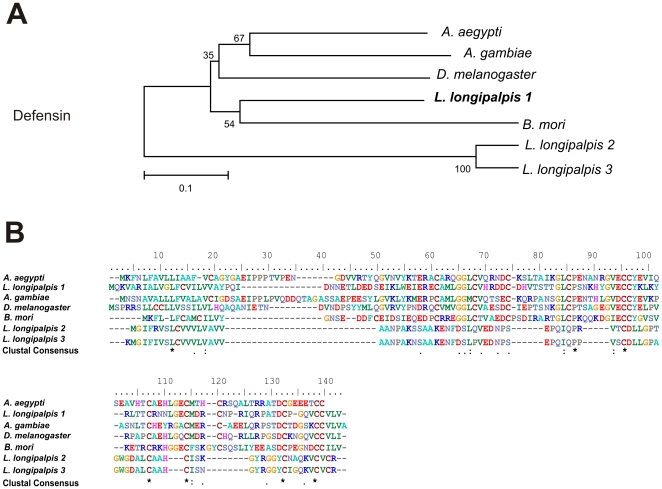
β-defensin sequence analysis. (A) Neighbor-joining tree of putative β-defensins: *L. longipalpis* 1 (BAR005E03/AM091821, male reproductive organs and whole female cDNA libraries), *L. longipalpis* 2 (EU124626, midgut female library) and *L. longipalpis* 3 (EX211140, midgut female library), *A. aegypti* (AEL009861), *A. gambiae* (AGAP007049), *D. melanogaster* (CG10433), and *B. mori* (NP_001106745). Bootstrap percentage values indicated in nodes are based on 1000 replicates. (B) Multiple alignment of putative β-defensin of male reproductive tracts from *L. longipalpis* and its orthologues in Diptera. Conserved amino acids are indicated by (*).

In *L. longipalpis*, defensins were also identified in a female midgut cDNA library [14], [15]. However, these sequences are quite different from the one we found, which is more closely related to other putative defensins found in the reproductive tracts from other Diptera (Figure 3).

**Figure 4 pone-0034495-g004:**
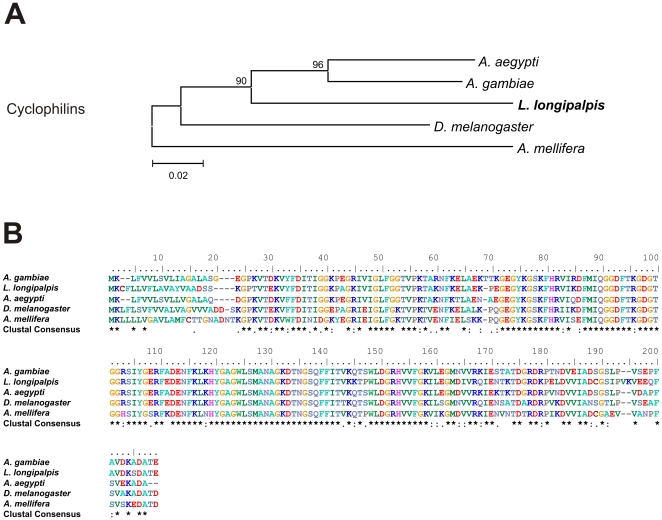
Cyclophilin sequence analysis. (A) Neighbor-joining tree of putative cyclophilin *L. longipalpis* (RAAPBAR022E08/AM092289, male reproductive organs and whole female cDNA libraries), *A. gambiae* (AGAP007088-PA), *A. aegypti* (AAEL013279), *D. melanogaster* (FBpp0071844/CG2852) and *A. mellifera* (NP_001229473). Bootstrap percentage values indicated in nodes are based on 1000 replicates. (B) Multiple alignment of putative cyclophilin of male reproductive tracts from *L. longipalpis* and its orthologues in Diptera. Conserved amino acids are indicated by (*).

Cyclophilins are another type of Acp related to the immune response. Also called immunophilins, they modulate the female's response to infection in *D. melanogaster* [38]–[40]. One of our ESTs (RAAPBAR22E08) shows homology to AAEL013279, one of the two cyclophilins described in *A. aegypti* [33]. Cyclophilins have been found in the reproductive tract of others insects [22], [30], [33]. The alignment of *L. longipalpis* cyclophilin and some of its insect homologues show a large region of identity, especially with mosquito ciclophilins (Figure 4)

### Fibrinogen and fibronectin proteins

The *L. longipalpis* sequence RAAPBAR013A01 has shown homology to the putative *A. aegypti* mRGPs (AAEL001713), that belongs to the fibrinogen/fibronectin protein family and could possibly be involved in blood coagulation and digestion [33], [41]–[43]. Although, the release of some Acps from the reproductive tract into the hemolymph has not been reported in mosquitoes yet [33], this possibility cannot be discarded since it has been observed in *D. melanogaster* [31], [44]. However, in *A. gambiae*, a protein that belongs to this family (AGAP07041) and is exclusively expressed in male accessory gland was found in the mating plug [32].

### Hydrolases

Similarly to *A. gambiae* [22], two classes of hydrolases were found in *L. longipalpis* male reproductive organs: lipases and carboxylesterases. The sequences RAAPBAR018E11 and RAAPBAR031D02 showed similarity to lipases while RAAPBAR013H06 has shown similarity to carboxylesterase genes from *A. gambiae* (AGAP005370-PA, COEBE4D). Lipases hydrolyze triglycerides and provide energy to sperm [45]. COEBE4D was described in *A. gambiae* as a *D. melanogaster* esterase 6 homologue (EST-6), which is expressed in male genitalia. EST-6 is known to influence egg-laying behavior and receptivity to re-mating, when semen is transferred to the female during mating [46].

### Proteolysis-related ESTs

In insects, proteases and protease inhibitors are associated to very diverse biological processes including, among them, post-mating changes in female physiology, such as ovulation and sperm storage [31]. Proteases and protease inhibitors correspond to the second and third most frequent Acp classes in *D. melanogaster*, respectively [30]. In addition, proteases represent 25% of *A. aegypti* mRGPs [33] and have been found in mating plug of *A. gambiae* [32]. Three ESTs identified as putative *L. longipalpis* male mRGPs showed homology to proteases, one serine protease and two metalloproteases (Table 4). The EST RAAPBAR030E12 showed similarity to AAEL006576, one of the seven serine proteases predicted in the male reproductive gland of *A. aegypti* [33].

**Table 4:** ESTs with other specific function.

Cluster name	N	Program	Database	e-value	Homology
RAAPBAR27H05	3	Blastx	Uniprot	1.0e-43	Cytoplasmic Dynein light chain
RAAPBAR014D07	1	Blastx	AaegL1.2_vectorbase	8.0e-42	AAEL013018 (general OBP)
RAAPBAR019E07	1	Blastx	AaegL1.2_vectorbase	2.0e-25	AAEL000071 (OBP 56a)
RAAPBAR019E09	3	Blastx	CquiJ1.2_vectorbase	7.0e-22	CPIJ012719-PA (OBP 56d)

**N-** Number of reads. OBP-Odorant Binding Protein.

**Figure 5 pone-0034495-g005:**
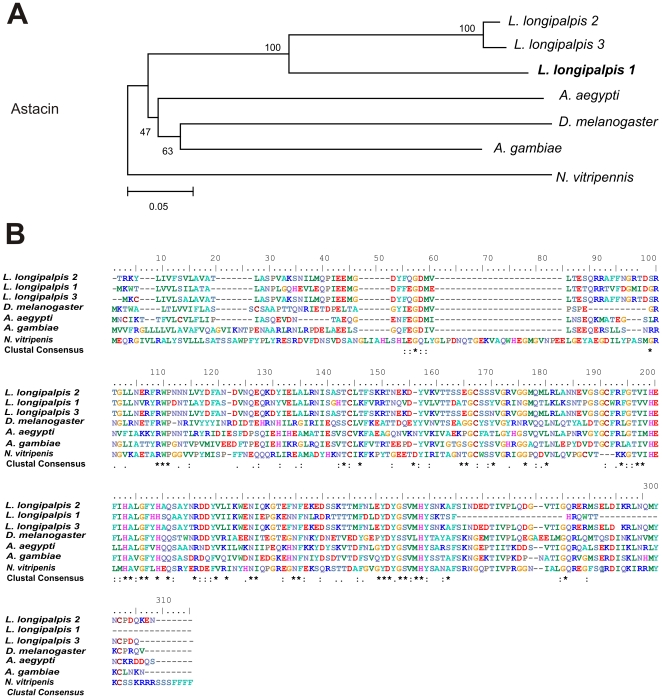
Astacin metalloprotease sequence analysis. (A) Neighbor-joining tree of putative astacin from *L. longipalpis* (RAAPBAR022F08 male reproductive organs cDNA libraries), *L. longipalpis* 2 (AM088883 whole female cDNA libraries) and *L. longipalpis* 3 (Lulo-Astacin A8CW49_LUTLO, midgut female library) *A. aegypti* (AAEL013449), *A. gambiae* (AGAP010764), *D. melanogaster* (FBpp0080341/CG15254) and *Nasonia vitripenis* (NV12552). Bootstrap percentage values indicated in nodes are based on 1000 replicates. (B) Multiple alignment of putative astacin of male reproductive tracts from *L. longipalpis* and its orthologues in Diptera. Conserved amino acids are indicated by (*).

The ESTs RAAPBAR008G04 and RAAPBAR022F08 showed similarity to proteases from the metalloprotease family. RAAPBAR022F08 presented homology to AAEL013449, one protease found in the reproductive tract of mated females of *A. aegypti* and absent in virgin females [33]. However the best observed similarity was a zinc-dependent metalloprotease astacin-like of *A. gambiae* (AGAP010764-PA). In *D. melanogaster*, the astacin CG11864 is synthesized in the male accessory gland and is processed into a smaller form when it crosses the male reproductive tract on its way to the female. This cleavage creates an active astacin that seems to be involved in the processing of other Acps (Acp26Aa and Acp36DE) in the female tract [47]. In *L. longipalpis*, a zinc-metalloprotease (A8CW49_LUTLO) was identified as a likely astacin in a midgut female cDNA library [14]. This astacin shows high similarity to the EST AM088883 from *L. longipalpis* whole body female cDNA library [11]. Figure 5 shows a neighbor-joining tree constructed using the three ESTs of *L. longipalpis* and astacin sequences of other insects.

**Figure 6 pone-0034495-g006:**
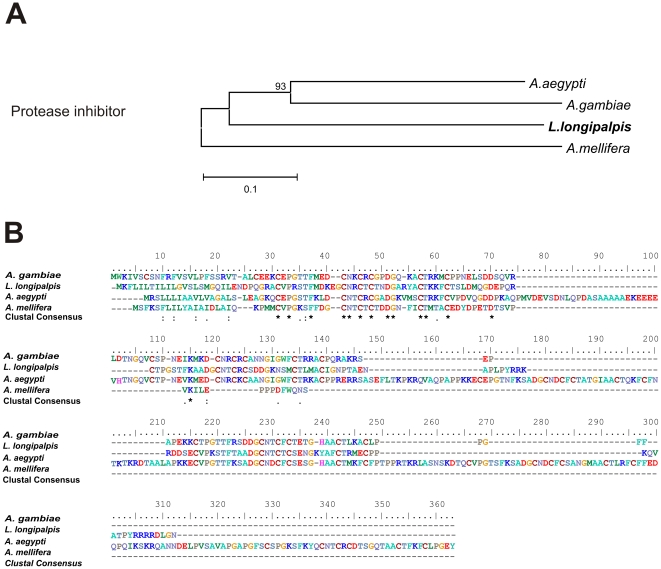
Protease inhibitor sequence analysis. (A) Neighbor-joining tree of putative protease inhibitor *L. longipalpis* (RAAPBAR023H02/EW989852 B male reproductive organs and midgut female cDNA library), *A. aegypti* (AAEL000551), *A. gambiae* (AGAP011319), and Apis mellifera (XP_003250953). Bootstrap percentage values indicated in nodes are based on 1000 replicates. (B) Multiple alignment of putative protease inhibitor of male reproductive tracts from *L. longipalpis* and its orthologues in Diptera. Conserved amino acids are indicated by (*).

Protease inhibitors presenting serpin domains correspond to 11% of predicted *A. aegypti* mRGPs [33]. Eight serpin genes were found in *A. gambiae*, seven in *A. aegypti* [33] and seven in *D. melanogaster* [40]. In *L. longipalpis* two essentially identical ESTs, one from our library (RAAPBAR023H02) and one from a midgut library (EW989852), show high similarity to a serine protease inhibitor member of the pacifastin family. This kind of protease inhibitor is only found in arthropods [48]. Members of this protein family present two heterodimeric chains (heavy and light) with different biological roles. The serine peptidase inhibitory activity is found in ‘Pacifastin Light Chain Domains’. Among the seven serpin genes found in *A. aegypti*, only one (AAEL000551) has a pacifastin inhibitor domain [33], [49] and presents high similarity to the *L. longipalpis* sequence (Figure 6). Pacifastins have also been shown to be possible modulators of the prophenoloxidase pathway, potentially implicating them in the insect immune response [48].

### Cysteine Rich Secretory Protein (CRISP)

Only one EST (RAAPBAR022H02) has shown similarity to a predicted *A. aegypti* CRISP (AAEL009239), which is highly similar to a gene expressed in the salivary glands belonging to a family of proteins (Antigen-5) found in several blood-feeding arthropods [33]. CRISPs are found in *D. melanogaster* seminal fluids [47] and, in *A. aegypti* reproductive glands [33]; these proteins are involved in sperm–egg interactions [30].

### ATP synthase

The candidate *L. longipalpis* mRGP, RAAPBAR005C10, shows homology to AAEL007777, a vacuolar *A. aegypti* ATP synthase, also described as an mRGP [33]. Recently, this protein was found in the seminal fluid proteome of *A. aegypti*, as one of the proteins that are transferred to females but do not present a signal peptide sequence [34].

### ESTs associated with protection from oxidative stress and protein folding

The ESTs RAAPBAR020D12 and RAAPBAR011H02 showed similarity to thioredoxin proteins family (Table 3). The RAAPBAR020D12 presented similarity to AAEL010777-RA of *A. aegypti* and AGAP009584, a thioredoxin that is expressed in the male accessory glands of *A. gambiae* and is present in its mating plug [32]. Although RAAPBAR020D12 also showed similarity to the *D. melanogaster* Acps CG6988, a predicted protein disulfide isomerase (PDI) that also presents a thioredoxin domain [45], analysis indicates that they are not orthologous. RAAPBAR011H02 showed similarity to AGAP0072001 of *A. gambiae* and also to AAEL000641, one of the eight *A. aegypti* mRGPs involved in protein folding.

**Figure 7:**
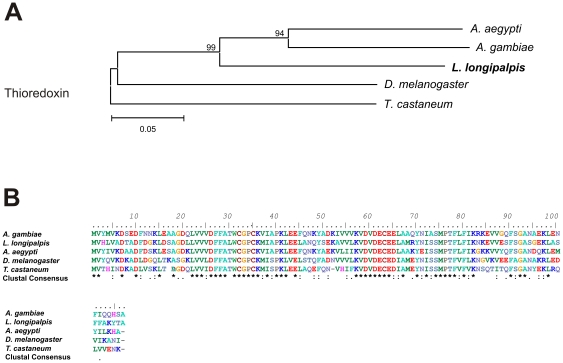
Thioredoxin sequence analysis. (A) Neighbor-joining tree of putative thioredoxin *L. longipalpis* (RAAPBAR020D12 male reproductive organs cDNA libraries), *A. aegypti* (AAEL010777), *A. gambiae* (AGAP009584-PA) and *Tribolium castaneum* (XM_962894.2). Bootstrap percentage values indicated in nodes are based on 1000 replicates. (B) Multiple alignment of putative thioredoxin of male reproductive tracts from *L. longipalpis* and its orthologues in Diptera. Conserved amino acids are indicated by (*).

Thioredoxins belong to an antioxidant class of proteins involved in protection from oxidative stress [50]. It is likely that the *L. longipalpis* thioredoxin is involved in protecting sperm and/or the reproductive tract in mated females against oxidative damages, as previously proposed for *D. melanogaster* [30] and *A. aegypti* [33]. Figure 7 shows a neighbor-joining tree comparing RAAPBAR020D12 to other orthologous insect thioredoxins. As expected the *L. longipalpis* putative thioredoxin shows higher similarity to the mosquito sequences. A similar analysis of RAAPBAR011H02 was not carried out as it seems to be incomplete.

### ESTs with other specific functions

In addition to the putative mRGPs mentioned above, the comparison between our ESTs and different databases identified few other sequences related to specific functions of particular interest (Table 4). One EST shows similarity to dynein, a protein involved in cellular processes during sperm individualization. Dynein is enriched around spermatid nuclei during post-elongation stages [23].

Three ESTs (Table 4) show homology to odorant binding proteins (OBPs). Insect OBPs are small proteins present in the olfactory system [51] and perform an important odor-specific function in olfaction by interacting with subsets of odorant molecules. The *D. melanogaster* OBPs are expressed in the olfactory and gustatory sensilla [52], but some were also found in *A. aegypti* male reproductive gland proteins [33].

The ESTs RAAPBAR014D07 and RAAPBAR19E07 showed similarity to two classical OBPs named general OBP and OBP56a of *A. aegypti*, respectively. Although some OBPs have been found in the reproductive system of *A. aegypti*, they are not the homologues of those found in *L. longipalpis*. Chemical communication is very important in reproduction and the presence of classical OBPs in *L. longipalpis* male reproductive organs could be related to chemical interactions during mating. However the possibility that these OBPs are related to other functions cannot be discarded [30].

### Other ESTs encoding potential Male Reproductive Gland Proteins (mRGPs)

Recently, Sirot et al [34] identified 93 proteins from seminal fluid (Sfps) that are transferred to *A. aegypti* females during mating. Comparing the list of these proteins with the BLAST results of our EST library against *A. aegypti* peptides (Table S3), we identified 11 additional *L. longipalpis* sequences, as shown in Table S7. These sequences encoding different protein classes therefore represent other potential *L. longipalpis* mRGPs.

Finally, in another recent paper, Baker et al [53] published a gene expression atlas of sex- and tissue-specificity in *A. gambiae* which includes a list of genes with increased expression levels in the male accessory glands. We compared this list with the *L. longipalpis* ESTs that have shown hits against the *A. gambiae* protein databank (Table S5). A total of 65 *L. longipalpis* sequences were identified which are listed in Table S8. Since their *A. gambiae* homologues present increased levels of expression in male accessory glands these *L. longipalpis* sequences probably include a number of other potential mRGPs.

### Conclusions

A large number of sequences encoding diverse protein families were identified in the transcriptome of *L. longipalpis* male reproductive system. Several ESTs, however, did not fall into any predicted proteins from insects or other arthropods. A number of the identified ESTs are similar to mRGPs/Acps of *A. aegypti*, *A. gambiae* and *D. melanogaster*. Like in other insects, these proteins are probably involved in important aspects of sand fly reproductive biology, such as female post-mating behavior and physiology. They have also a potential role in sexual selection and speciation. Therefore the data we generated might be very useful for further molecular and evolutionary genetic studies of sand flies. In addition, as suggested by a number of other authors [22], [25], [30], [31]–[34], [53] the identification of uniquely expressed genes, particularly those involved in reproduction, may serve as potential knockout targets for future methods of control involving different forms of vector genetic manipulation. Hence, it is possible that some of the sequences we identified might become useful in the future control of sand fly vectors of leishmaniases, a group of very important but neglected tropical diseases.

## Materials and Methods

### mRNA Extraction, Library Construction and Sequencing

The male reproductive organs (testes, vasa deferentia, seminal vesicle, ejaculatory duct and external genitalia, see [26] for details) of adult *L. longipalpis* males from Lapinha Cave (Lagoa Santa, Minas Gerais State, Brazil) were dissected in RNA stabilization solution (RNA later™/QIAGEN) and then frozen at −80°C. The mRNA was isolated from about 200 male reproductive organs using the “QuickPrep™ Micro mRNA Purification” kit (Amersham Biosciences).

The cDNA synthesis and library construction were carried out according to the “Creator™ SMART™ cDNA Library Construction Kit User Manual” (Clontech). First strand cDNA was synthesized from 0.3 µg of mRNA in a 10 µl reaction according to the manufacturer's protocol. Two µl of first-strand cDNA were used to carry out ds cDNA synthesis by 23 cycles of PCR (95°C for 5 s, 68°C for 6 min) and the ds cDNA was digested with proteinase K and purified with phenol: chloroform: isoamyl alcohol. The purified ds cDNA was digested with the Sfi I enzyme and fractionated by a CHROMA SPIN-400 column. Finally, the Sfi I-digested cDNA was ligated into the pDNR-LIB vector and transformed in DH5α (Library Efficiency DH5α Competent Cells/Invitrogen) competent cells. cDNA clones were sequenced using the “Big Dye Terminator v3.1 Cycle Sequencing kit” (Applied Biosystems) with the M13 forward and T7 primers. Reactions were run on an “Applied Biosystems 3730 DNA Analyzer” (Applied Biosystems).

### Sequence Analysis

Sequences were edited and analyzed with the program GARSA [54]. Reads were vector and quality trimmed using the phred/phrap package (http://www.phrap.org/phredphrapconsed.html) incorporated into STINGRAY (http://stingray.biowebdb.org), a newer system for sequence analysis built on the original GARSA. Reads were considered to have low quality when: the length was below 100 bp, the “N” percentage was greater than 1% throughout the sequence and/or a large portion of vector sequence was found (before and after the insert). Only high quality reads (phred >20) were assembled into clusters by GARSA using CAP3 following the parameters (-o 25, -b 20, -d 200, -p 95) and were compared with several databases using Blast programs. After that, all sequences were submitted to SignalP version 3.0. Preliminary annotation was done using the 3 ontologies of the Gene Ontology Consortium (http://www.geneontology.org).

Sequences of mRGPs/Acps described in *D. melanogaster* [30], *A. gambiae* [22] and *A. aegypti* [33] were used for custom database construction with the program formatdb. This customized database was used to find male reproductive gland proteins (mRGPs) in the transcriptome of *L. longipalpis* male reproductive organs by similarity using the Blast programs. To confirm these results we used a two-step Blast approach where the best match of each Acp was considered.

Sequence alignments of translated nucleotide or amino acid sequences and their associated Neighbor-joining trees show in Figures 3–7 were performed with Clustal X version 2.0 [55] and MEGA 4 [56], respectively.

## Supporting Information

Table S1 Best BLASTn results from the comparison between *L. longipalpis* EST database and the *L. longipalpis* cDNA library of male reproductive organs.
(XLS)

Table S2 Best BLASTx results from the comparison between RefSeq_protein database and the cDNA library of *L. longipalpis* male reproductive organs.
(XLS)

Table S3 Best BLASTx results from comparison between PEPTIDES of *A. aegypti* (AaegL1.2 database) and cDNA library of male reproductive organs of *L. longipalpis*.
(XLS)

Table S4 Best BLASTx results from the comparison between the dmel-all-translation-r5.35 database and the cDNA library of *L. longipalpis* male reproductive organs.
(XLS)

Table S5 Best BLASTx results from comparison between PEPTIDES of *A. gambiae* (AgamP3.6 database) and cDNA library of male reproductive organs of *L. longipalpis*.
(XLS)

Table S6 Best tBLASTx results from the comparison between Acps/mRGP-Anopheles-Drosophila-Aedes and the cDNA library of *L. longipalpis* male reproductive organs.
(XLS)

Table S7 *L. longipalpis* ESTs with homology to seminal fluid proteins of *A. aegypti*.
(XLS)

Table S8 *L. longipalpis* ESTs with homology to genes with increased expression levels in the male accessory glands of *A. gambiae*.
(XLS)
